# A review of the endocrine disrupting effects of micro and nano plastic and their associated chemicals in mammals

**DOI:** 10.3389/fendo.2022.1084236

**Published:** 2023-01-16

**Authors:** Sana Ullah, Shahid Ahmad, Xinle Guo, Saleem Ullah, Sana Ullah, Ghulam Nabi, Kunyuan Wanghe

**Affiliations:** ^1^ Centre of Biotechnology and Microbiology, University of Peshawar, Peshawar, Pakistan; ^2^ School of Ecology and Environment, Hainan University, Haikou, Hainan, China; ^3^ Academy of Plateau Science and Sustainability, College of Life Sciences, Qinghai Normal University, Xining, China; ^4^ Department of Zoology, Division of Science and Technology, University of Education, Lahore, Pakistan; ^5^ Institute of Nature Conservation, Polish Academy of Sciences, Krakow, Poland; ^6^ Key Laboratory of Adaptation and Evolution of Plateau Biota, Laboratory of Plateau Fish Evolutionary and Functional Genomics, Qinghai Key Laboratory of Animal Ecological Genomics, Northwest Institute of Plateau Biology, Chinese Academy of Science, Xining, China

**Keywords:** microplastics, nanoplastics, mammalian endocrine system, endocrine abnormalities, endocrine disrupting chemicals, plastic additives, environmental pollution

## Abstract

Over the years, the vaste expansion of plastic manufacturing has dramatically increased the environmental impact of microplastics [MPs] and nanoplastics [NPs], making them a threat to marine and terrestrial biota because they contain endocrine disrupting chemicals [EDCs] and other harmful compounds. MPs and NPs have deleteriouse impacts on mammalian endocrine components such as hypothalamus, pituitary, thyroid, adrenal, testes, and ovaries. MPs and NPs absorb and act as a transport medium for harmful chemicals such as bisphenols, phthalates, polybrominated diphenyl ether, polychlorinated biphenyl ether, organotin, perfluorinated compounds, dioxins, polycyclic aromatic hydrocarbons, organic contaminants, and heavy metals, which are commonly used as additives in plastic production. As the EDCs are not covalently bonded to plastics, they can easily leach into milk, water, and other liquids affecting the endocrine system of mammals upon exposure. The toxicity induced by MPs and NPs is size-dependent, as smaller particles have better absorption capacity and larger surface area, releasing more EDC and toxic chemicals. Various EDCs contained or carried by MPs and NPs share structural similarities with specific hormone receptors; hence they interfere with normal hormone receptors, altering the hormonal action of the endocrine glands. This review demonstrates size-dependent MPs’ bioaccumulation, distribution, and translocation with potential hazards to the endocrine gland. We reviewed that MPs and NPs disrupt hypothalamic-pituitary axes, including the hypothalamic-pituitary-thyroid/adrenal/testicular/ovarian axis leading to oxidative stress, reproductive toxicity, neurotoxicity, cytotoxicity, developmental abnormalities, decreased sperm quality, and immunotoxicity. The direct consequences of MPs and NPs on the thyroid, testis, and ovaries are documented. Still, studies need to be carried out to identify the direct effects of MPs and NPs on the hypothalamus, pituitary, and adrenal glands.

## 1 Introduction

xIn recent decades, plastic pollution has become one of the most widespread and enduring anthropogenic alterations in all environmental compartments of our planet’s surface (1) and therefore, considered as a stratigraphic marker for the Anthropocene (2). A culture of single-use plastic, rapid and inexpensive plastic production and non-circular economic models led to the creation of over 368 million metric tons (Mt) of single-use plastic in 2019 (3). It is expected that if the current production and waste management trends continue, approximately 12,000 Mt of plastic waste will end up in the natural environment by 2050 (4). To date, plastic debris has affected 3876 species only in the aquatic environment (https://litterbase.awi.de/interaction_detail; date assessed July 28, 2022), and by the year 2050, plastic will be found in the digestive tract of 99% of all sea bird species (5). Globally mammals are already at-risk due to several reasons, including climate change (6), however, bioaccumulation of the microplastic (MP) and associated toxic chemical additives are accelerating the risk of extinction (7, 8). Despite its ubiquitous distribution, current knowledge about the health effects of MP and associated chemicals in mammals is limited. Therefore, we aim to highlight how MP affects mammalian endocrine glands, which could contribute to their conservation and management, especially in vulnerable populations.

### 1.1 Rising global plastic and MPs pollution in the terrestrial and aquatic environment

Plastic waste is a contemporary societal and ecological issue due to its indispensable nature and ubiquitous use in daily life, associated with long-term detrimental effects on organisms (9). Plastic consumption will continue to rise in response to global population growth, and as plastic degrades, microplastics (<5 mm) and nanoplastics (< 1 nm) enter the terrestrial ecosystem in several ways (10). Depending on their manufactured and fragmented origin, MPs can be classified as primary or secondary (11). Primary MPs are primarily designed into small sizes for commercial practices like personal care products, whereas secondary MPs are fragmented from larger plastics by various physical and biological methods (10), such as UV radiation, temperature changes, and wave action (12). Due to sewage sludge applications, each year, 63–430 and 44–300 thousand tons of MPs are added to agro-ecosystems in Europe and North America (13). In marine ecosystems, approximately 5-13 million tons of plastic debris enter the ocean each year (8). Consequently, the world’s upper ocean currently comprises 24.4 trillion pieces, (8.2 × 104 ~ 57.8 × 104 tons) of micro-plastic (14), and its concentration might exceed 250 million metric tons by 2025 (8, 15). In 2015, Oceanographers estimated 15-51 trillion MP particles floating on water surfaces worldwide (16). In Europe, approximately, 63,000–430,000 tonnes of MPs entered the farmlands annually (17), while in most part of the world, data regarding MP loading in agriculture farmlands are unavailable.

### 1.2 Chemical composition of MPs and their endocrine-disrupting effects

**Figure 1:**
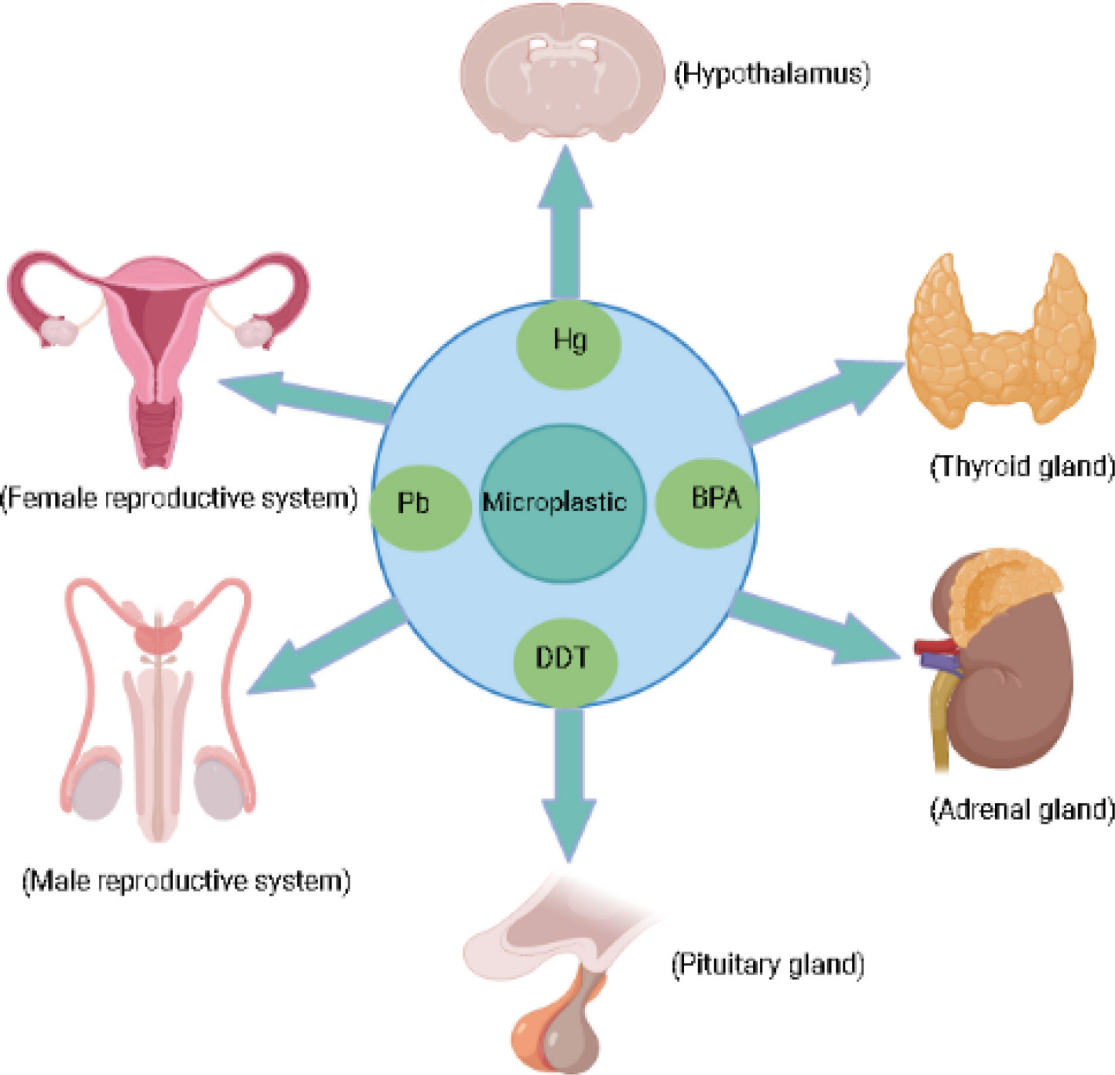
Micoplastic and their associated chemicals exposure can effect endocrine glands.

MPs generated from plastic degradation persist for hundreds and thousands of years in the environment (18). Plastic bottles, disposable diapers, and polystyrene foam have a life span of 450, 500, and >5000 years [https://www.goecopure.com/lifespan-of-plastic.aspx]. Plastic additives used during plastic processing contribute up to 70% of plastics (18). More than 10,000 chemicals are identified as plastic additives, and 2400 chemicals have been classified as detrimental to marine and terrestrial biota (19). These additives include plasticizers, antioxidants, UV stabilizers, dyes, and flame retardants. Some of them are of serious concern, such as alkylphenol (20), polybrominated diphenyl ethers [PBDEs] (21), phthalates, organotins, perfluorinated compounds, dioxin (22), bisphenol A [BPA] and heavy metals like chromium, lead, and cadmium (8, 23). Approximately, 1000 chemicals classified as endocrine disruptor chemicals (EDCs) alter the expression of various hormone receptors and interfere with the synthesis, secretion, transport, and action of hormones, leading to endocrine and developmental abnormalities (23, 24). Nearly nine different forms of MPs are reported in human feces from multiple countries, clearly validating the presence of MPs in the human food chain and warning us about their harmful effects on human health (25). MPs and their composite toxic additives can cross various biological membranes, blood-brain barriers and both can interfere with various hormone receptors, thereby disrupting different hypothalamic axes such as the hypothalamic-pituitary-thyroid axis [HPT], the hypothalamic-pituitary-adrenal axis [HPA], and hypothalamic-pituitary-gonadal axis (HPG; **Figure 1**) (23, 26–29). Various metabolic disorders, gut dysbiosis, and intestinal barrier dysfunction induced by MPs have been explored using rats and mice as model organisms. Similarly, neurobehavioral changes, disrupted thyroid status, and biochemical stress are the direct consequences of MPs exposure in rats (30).

### 1.3 MPs bioaccumulation and biomagnification due to their “Trojan Horse” effects in mammals

The large surface area and hydrophobic surface of MPs make them a suitable medium for carrying many pollutants such as EDCs, heavy metals, and other toxic organic chemicals, making them harmful to mammals through bioaccumulation and biomagnification processes (31, 32). These are called “Trojan Horse Effects” of MPs (33), and induce several synergistic, behavioral, histological, and biomolecular alternations (32). Many EDCs and other pollutants are added as additives or absorbed by MPs; after being consumed directly or indirectly through the food web, MPs increase their bioaccumulation in mammals (8, 21, 34). MPs accumulate in various body parts and are involved in biochemical pathways, affecting cell functioning by crossing biological membranes in a size-dependent manner (35). Studies have shown that MPs of size 0.1-10 µm can cross biological membranes, blood-brain barrier, and even placenta, enhancing the possibilities of their bio-accumulation in secondary tissues such as the liver and brain (23). While MPs <150µm can cross the gastrointestinal tract, those <5µm can accumulate in macrophages and be carried to the blood circulation and the spleen (22). Similarly, MPs <10µm trans-locate from the gut to the circulatory system and can accumulate in the liver, kidney, and brain (36).

This review warns about the accumulation of MPs in marine and terrestrial ecosystems. Furthermore, it implies that studies need to be carried out to deeply understand the action mechanism of MPs, nanoplastics [NPs], and associated chemicals in aquatic and terrestrial biota and their long-term detrimental consequences on the mammalian endocrine system.

## 2 Effects of MPs, NPs, and associated chemicals on the mammalian thyroid gland

The thyroid gland is an essential endocrine gland responsible for normal brain function, growth, and neurological development of all animals (37). The thyroid functions under the HPT axis and affects almost every organ in the body (38), therefore disruption in thyroid homeostasis can be detrimental and will affect the body’s overall health status. Long-term exposure to plastic particles and associated chemicals has been shown to exhaust thyroid endocrine function by weakening its driving forces in regulating growth, development, metabolism, and reproduction (39). MPs additives and pollutants, such as PBDEs, BPA, phthalates, and organotin act as thyroid-disrupting chemicals [TDCs] (22) (**Table 1**). Similarly, MPs cause thyroid dysfunction and developmental abnormalities once ingested with associated POPs and EDCs (43). Phthalate causes a reduction in thyroid weight during childhood exposure and associated with developmental abnormalities and hyperactivity of the thyroid gland (44, 45). These TDCs associated with plastic enter the body through the gastrointestinal tract and interfere with T4 and T3 biochemical pathways, while their circulation adversely affects thyroid hormone production and metabolism, affecting other organs like the brain in primary developmental stages (46). Several TDCs circulating in the blood form complexes with protein of thyroid hormones and eventually reach the brain and bind with thyroid hormone receptors, disrupting thyroid health (46). TDCs are also responsible for the prevalence of subclinical thyroid conditions known as “subclinical thyroid disease” [SCTD]. In SCTD, the body observes abnormal low or high levels of thyroid-stimulating hormones [TSH] (47). Similarly, BPA can interfere with thyroid hormone action and impairs thyroid functions by inhibiting T3 binding to its receptor and suppressing transcriptional activity mediated by thyroid hormone receptors (48). Phthalates also disturb the normal thyroid system by interfering with gene expression in the HPT axis and metabolic activity (27). It impairs thyroid function through various mechanisms, including inhibition of T3 protein binding, antagonistic interactions, and disruption of throid receptor’s transcriptional activity (49).

**Table 1:** The effects of MPs on the mammalian thyroid gland.

Plastics	Species	Thyroid disrupting consequences	References
MPs	Humans	Thyroid dysfunction and metabolic and developmental abnormalities once ingested with associated POPs and EDCs	(40)
NPs	Rats	T3 and circulating THs levels were decreased after exposure to PS NPs, while TSH significantly increased.	(41)
MPs	Rats	Remarkable lesions Ectopic thymus Ultimobranchial cyst.	(42)
MPs	Rats	Increased level of T3, FT3/FT4 ratio, and decreased level of TSH	(41)

PBDEs and polybrominated biphenyl [PBBs] as flame retardants in plastic (50) can decrease the circulating level of thyroid hormones and are associated with impaired thyroid function (51). It has been reported that five weeks of rats’ exposure to 1, 3, 6, and 10mg/kg/day of polystyrene nanoplastics [PS-NPs] suppress the serum level of T3, FT3, and FT4 synthesis and circulating level of thyroid hormones (41) (**Table 2**). Few researchers have demonstrated phthalates association with altered FT4 and total T3 in pregnant women (53). It has been shown that BPA and phthalates cause endocrine toxicity at all levels in animals (45).

**Table 2:** The MPs additives effects on the mammalian thyroid gland.

Endocrine disrupters	Species	Thyroid disrupting consequences	Reference
Phthalates	Humans	Thyroid epithelial cell hypertrophy and hyperplasia Thyroid hyperactivity, gene expression disruption of the hypothalamic-pituitary-thyroid [HPT] axis, thyroid antagonistic interaction, altered FT3 and FT4	(52) (44) (27) (49) (53)
Bisphenol A [BPA]	Rats	Inhibits T3 receptor binding ability, thyroid antagonist, thyroid oxidative damage	(48, 54)
Polybrominated diphenyl ethers [PBDEs]	Rats, Humans	Serum T4 reduction, the prevalence of hypothyroidism, disturb T4 levels in umbilical-cord blood, altered T3 and T4 levels	(51, 55) (56)
Tributyltin [TBT]	Rats	Dysregulated HPT axis, thyroid follicle reduction, decreased FT4 level	(57)
Polychlorinated Biphenyls (PCBs)	Rats	Reduced TT4 and FT4 levels	(58)
Hexabromocyclododecane (HBCD)	Rats	Thyroid follicular cell hypertrophy, reduced concentration of serum T3	(59)
Mercury	Humans	Contribute to thyroid cancer, hypothyroidism, and autoimmune thyroiditis	(60)
Dichlorodiphenyltrichloroethane [DDT]	Rats	Reduced T4 level and decreased size of follicles	(61)

Thyroid hormone levels in pregnant rats and their progeny are altered by brominated flame retardant chemicals, resulting in obesity, heart illness, early puberty, and insulin resistance in their children (62). The PBDEs and PBBs can dissolve in lipids and fats, so their accumulation is easy in wildlife and marine animals once exposed to these chemicals (21). PBDEs reduce the circulating levels of thyroid hormones through changes in T4 binding, and reduction in serum T4 (55). PBDEs disrupt the HPT axis (63) by altering the gene transcriptions of multiple genes like thyroid stimulating hormone subunit [tsh], deiodinase type 2 [deio2], and NK2 homeobox 1 [nkx2.1] (64). These genes, regulating thyroid development and TSH synthesis, are extremely sensitive to PBDEs (64).

## 3 Effect of MPs, NPs, and associated chemicals on the male reproductive system

Due to the small size of MPs, they can easily enter the organism’s reproductive cells, tissues, and organs altering normal morphology, histology, and physiological functions of the male reproductive system (65). In recent years MPs’ toxicity to the reproductive system got much more attention because they have caused widespread male reproductive abnormalities in mice making them a potential hazard (66) (**Table 3**). Currently, this environmental issue with respect to mammalian reproductive health is poorly understood (65). However, the harmful effects of MPs on mice’s reproductive health might provide new insight into the deleterious effects of MPs on the mammalian reproductive system.

**Table 3:** MPs effects on the mammalian male reproductive system.

Plastics	Species	Consequences on male reproductive system	References
MPs	Mice	Recused sperm quality, abnormal testicular spermatogenesis	(67)
MPs	Mice	Testicular transcriptomic alterations, altered spermatogenesis	(68)
MPs	Mice	Decreased testosterone levels, disruption of Blood Testes Barrier [BTB], testicular inflammation	(66)
MPs	Swine	Increased apoptosis and necrosis in testes, decreased viability of testicular cells	(69)
MPs	Mice	Decreased testicle weight and sperm quality, altered sperm phenotype	(70)
MPs	Rats	Damaged seminiferous tubule, destruction of BTB, spermatogenic cell apoptosis	(71)
PS-MPs	Mice	Oxidative stress in testes reduced sperm motility	(72)

The limited preliminary studies have suggested MPs bioaccumulation in mammalian testes with subsequent adverse reproductive outcomes (65). MPs ≤10 μm have been observed to accumulate in mice testes reducing testosterone [T] concentration, sperm quality and causes testicular inflammation (66). It also penetrates the testicular tissues such as Leydig cells, germ cells, and Sertoli cells (66). The accumulation of MPs in these cells may profoundly contribute to our understanding of MPs’ effects on mammalian reproductive health.

MPs contaminated with phthalate esters [PAEs] accumulated in the testes and altered testicular weight and sperm physiology by reducing sperm number and vitality (68). MPs cause morphological alternation in sperm like loss of sperm acrosome, cephalic having a small head, acephalia having no head, and tailless sperm (66). Exposure to these plastic particles at developmental stages resulted in shrank germ cells and decreased sperm density in the seminiferous tubules (73). MPs also induces irregular rearrangement of spermatid in testicular seminiferous tubules reducing spermatids’ number (67). A recent study (67) suggested PS-MPs reduce sperm production in mice through testicular injury. Deng et al. (68) observed spermatogenic disruption through altered acid phosphatase [ACP], superoxide dismutase [SOD], and malonaldehyde [MDA] levels in testes. ACP present in Sertoli cells (74) providing structural and nutritional support to spermatogenesis, were significantly increased while SOD and MDA levels were also increased inducing oxidative stress in testes (68). Oxidative stress is the key factor responsible for male infertility due to the increased cell division rate and mitochondrial oxygen consumption in testicular tissues (75). PS-MPs not only cause spermatogenic defects and testicular abnormalities but also penetrate the blood-testis-barrier [BTB] (66) in Sertoli cells and accumulate in the organism testes (30). According to (67) mice exposed to 5 µm of MPs shows a reduction in sperm activity and testicular tissue damage by activating the p38 mitogen-activated protein kinases [MAPK] pathway.

Spermatogenesis is an essential differentiation mechanism that requires T secretion, nutritional support provided by the sertoli cell, and a suitable environment for the production of sperm protected by BTB (66, 76). Following exposure to MPs, the T secretion declined and causes BTB disruption (66) with dysregulated testicular spermatogenesis (67). MPs enter testicular tissues by disrupting BTB, which serves as a physical barrier preventing the penetration of toxic chemicals to the testis, thus providing a healthy environment for spermatogenesis (66, 77) (**Table 3**). Due to BTB disruption, some proteins expression linked with BTB like Claudin 11, N-Cadherin, Connexin and Occludin were significantly reduced (71). Excess bioaccumulation of MPs induces increased germ cell apoptosis and disrupted spermatogenesis by causing abscission and irregular arrangement of spermatogenic cells (72) and sperm DNA fragmentation; a primary factor responsible for reproductive impairment (30). In mice, MPs exposure resulted in the shedding of spermatogenic cells and the structural disruption of seminiferous tubules (71).

The development and regulation of the reproductive system depend on the HPG axis, which alludes to the connection between the hypothalamus, pituitary, and gonads (78). Reproductive regulation initiates at the hypothalamus level due to gonadotropin-releasing hormone (GnRH) secretion by neurosecretory cells to the hypothalamic-hypophysial system (79). In response to GnRH secretion, pituitary releases FSH and LH that control gonadal functions (79). In males, the HPG axis is responsible for T secretion and regulation of spermatogenesis (80). MPs disrupt the HPG axis (26) as their exposure in male mice reduces the serum concentration of FSH, LH, and T while estradiol level significantly increases (81). Therefore the reproductive abnormalities caused by MPs due to HPG axis disruption include delayed gonadal maturation and the altered ratio of sex hormones that hindered reproductive development (26).

Some studies have shown PS-MPs increase reactive oxygen species [ROS] in male zebrafish liver and gonads. Their exposure to MPs increases apoptosis in testes, affecting gamete production (82) and interact with plasma membrane permeability of gametes, preventing gamete binding and offspring growth (26). In zebrafish testis, silver nanoparticles induce increased cell apoptosis due to overexpression of apoptotic genes like BAX, caspase-3 and caspase-9 (83). MPs also cause the thickness of the basement membrane of the zebrafish testis, due to which the production of spermatozoa is attenuated and undergoes the atrophy of seminiferous tubules (82).

BPA as essential plasticizers causes abnormal spermatogenesis, disruption of BTB, production of poor semen quality, and oxidative stress (84) (**Table 4**). BPA and BPS consumption alters T secretions and causes cell proliferation by interfering with many receptors (91). PBDEs as persistent flame retardants alter sperm DNA methylation by disrupting the hypothalamic-pituitary-testicular axis, affecting the functional ability of Leydig cells and spermatogenesis (86). MPs contaminated with phthalates can also cause oxidative stress in testes, change the sperm physiology (68), and their esters like dibutyl phthalate [DBP], diethylhexyl phthalate [DEHP], and diisopentyl phthalate [DiPeP] are recognized as anti-androgenic endocrine disrupters (85). Nonylphenol as a persistent pollutant inhibits steroidogenesis and alters enzyme localization like P450 aromatase, 3β-hydroxysteroid dehydrogenase and 17β-hydroxysteroid dehydrogenase in *Podarcis Siculus* (92). Similarly, DDT causes testicular injury and possible transgenerational effects on epigenomes and transcriptomes of future generations (93). Some anthropogenic EDCs such as cadmium [Cd] and lead [Pb] beyond a certain levels have a close association with infertility in mice and humans (94) (**Table 4**). Both Cd and Pb adversely alters the HPG axis that harms testicular tissues directly (40) and disrupt spermatogenesis, spermiogenesis, and steroidogenesis (95). Mice administration to varying doses of PSMPs for six weeks induced a significant increase in sperm deformities rate and a decrease in sperm motility (72). PSMPs also cause a reduction in mice’s serum T levels and decrease the functional activity of various enzymes involved in sperm metabolisms such as succinate dehydrogenase and lactase dehydrogenase (72).

**Table 4:** MPs additives effects on the male mammalian reproductive system.

Endocrine disrupters	Species	Consequences on male reproductive system	Reference
Phthalates	Rats and Mice	Oxidative stress in testes, altered sperm’s physiology, anti-androgenic effects	(68, 85)
BPA	Mice	Abnormal spermatogenesis, blood-testis-barrier [BTB] disruption, poor semen quality, DNA damage in sperm cells	(84)
PBDEs	Male	Dysregulated sperm DNA methylation, altered spermatogenesis	(86)
TBT	Syrian hamsters	Adverse steroidogenic enzymes activity, impaired testosterone production, defective spermatozoa	(87)
PCBs	Harbour porpoises	Decreased testes weight, Reduced sperm and spermatid numbers, small seminal vesicles	(88, 89)
Chromium, lead and Mercury	Mice, Rabbits	Leydig cell tumors, attenuates serum level of luteinizing hormone [LH], testosterone, follicle-stimulating hormone, testicular stroma	(90)

## 4 Effects of MPs, NPs, and their associated chemicals on the female reproductive system

Little is known about the harmful effects of MPs and NPs on the ovaries of mammals but studies conducted on rats and aquatic organisms may provide new insights into deadly consequences caused by plastic particles in ovaries. The harmful effects of MPs and their compound substances are linked to a dysfunctional female reproductive system (26) (**Table 5**).

**Table 5:** Microplastics effects on the mammalian female reproductive system.

Plastic	Species	Consequences on the female reproductive system	References
MPs	Mice	Oxidative stress in ovaries, decrease the number of ovarian antral follicles and malondialdehyde [MDA] levels in ovaries	(96)
MPs	Mice	Decreased pregnancies and increased mortality	(97)
MPs	Mice	Spontaneous abortion, decreased diameter of uterine arterioles, decreased uterine blood supply	(98)
MPs	Rats	Granulosa cell apoptosis, ovary fibrosis, and pyroptosis	(99)
MPs	Rats	Granulosa cells pryptosis through NLRP3/Caspase-1 signaling mechanism,	(100)

Research has shown MPs accumulation in rat’s ovaries and granulosa cells, reducing the growth of the follicles, decreasing the level of anti-Mullerian hormone [AMH] (99), estradiol, and causing an irregular estrous cycle and abnormal folliculogenesis (101). Granulosa cells are the essential somatic cells of the ovary responsible for normal ovarian development and maturation and play a significant role in folliculogenesis (102). In addition, PS-MPs also induce fibrosis of the ovary through activation of the Wnt/β-Catenin signaling pathway and apoptosis of granulosa cells by oxidative stress, reducing normal ovarian reserve capacity in rats (99, 103). The wnt/β-Catenin signaling pathway is essential for maintaining tissue homeostasis and regulating embryonic maturation, cell proliferation, and apoptosis (104).

MPs as a transport medium for their composite EDCs (105) induce various endocrine disorders like infertility, precocious puberty, hormone-based tumors, several metabolic problems, disruption of granulosa cell steroidogenesis, and polycystic ovary syndrome [PCOS] (106, 107) (**Table 6**). Endocrine disrupting plastic additives like PBDEs, BPA, phthalates, organotins (20), nonylphenols, octylphenols (113), and biocides like TBT, mercury, arsenic, copper, cadmium, and lead (114) can transfer from pregnant women to the fetal bloodstream through a placental barrier causing neurodevelopmental abnormalities in infants (115). In mice and monkeys, BPA disrupts oocyte development (116) and induces impairment and disruption of steroidogenesis in humans, ovine, swine (107), and murine granulosa cells (117). Several concerns have been raised demonstrating phthalates effect on granulosa cell steroidogenesis in humans (118), mice (108), and rats (119), and increase cell proliferation in swine when exposed to NPs with their composite EDCs (120). Similarly, cadmium disrupts gonadal steroidogenesis and inhibits the binding of FSH to its specific receptor, and alters steroid production of ovarian granulosa cells (121).

**Table 6:** The effects of MPs additives on the female mammalian reproductive system.

Endocrine disrupters	Species	Consequences on the female reproductive system	References
Phthalates	Mice	Reduced LH, defective ovarian steroidogenesis	(108)
BPA	Humans	Inhibiting secretion of progesterone and oestradiol, decreases the expression of CYP11A1	(107)
PBDEs	Humans	Increased menstrual cycle and bleeding time	(109)
TBT	Rats	Irregular ovarian adipogenesis, Ovarian fibrosis	(110)
PCBs	Mice	Follicular atresia, suppressed level of LH, and progesterone	(111, 112)
Chromium, lead and Mercury	Mice and Rabbits	Follicular astresia, low follicle growth, corpus luteum	(90)

Some aquatic studies have also shown the bioaccumulation of PS-MPs in female fish embryo yolk sacs and female eggs, affecting the normal physiology of offspring in female fish (122, 123). The ability of polystyrene MPs to interfere with plasma proteins connected to oocytes facilitates MPs’ cross-generational transfer (123). In addition, PSMPs delay ovarian development, decline the reproductive ability of marine medaka (26) and reduce superoxide dismutase [SOD], catalase [CAT], glutathione S-transferase [GST], and glutathione peroxidase [GSH-PX] in Oryzias melastigma ovary (99, 123). Similarly, MPs disrupt the HPG axis by down-regulating the transcription of genes like *GnRH, vitellogenin [Vtg]*, and choriogenin *[Chg]* in the steroidogenesis pathway (26) while its combined exposure with phenanthrene [Phe] increases the accumulation of Phe in the ovaries of marine medaka, disrupting ovarian development and the HPG axis (124).

## 5 Effects of MPs, NPs, and their additives on the hypothalamus

Hypothalamus is an essential part of the endocrine system that connects the nervous system to the endocrine system and secretes both inhibiting and releasing hormones that signal the pituitary gland to release various important hormones to the whole endocrine system (125). The detrimental consequences of MPs on the mammalian hypothalamus are poorly documented. However, there is clear evidence of mammalian hypothalamic-pituitary axes disruption caused by MPs and their composite EDCs that alter hormonal balance through feedback mechanisms (126, 127) (**Table 7**).

**Table 7:** MPs additives effects on the mammalian hypothalamus.

Endocrine disrupters	Species	Harmful effects	References
PBDEs	Rats	Dysregulation of HPT and hypothalamic-pituitary-gonadal [HPG] axis	(126)
BPA	Mice	Cause significant decrease in hypothalamic neurons, Cause astrocyte activation Impairs the function of proopiomelanocortin [POMC] neurons in the hypothalamic arcuate nucleus [ARC], Astrocyte-dependent hypothalamic inflammation	(128)
Phthalates	Rats	Dysregulation of the HPG axis, induce early puberty in female rats by inducing upregulation of hypothalamic IGF-1 expression, prolong the female estrous cycle, affects mRNA and protein expression of KiSS1, GPR54, and GnRH in the hypothalamus	(129)
PCBs	Rats	Oxidative stress in the hypothalamus, decreased hypothalamic weight, decreased acetylcholinesterase (AChE) activity	(130)
TBT	Rats	Disrupts the functional ability of the female HPG axis reducing some hormones like hypothalamic GnRH and decreasing secretion of pituitary LH. Distorting gene expression and provoking thyroid homeostasis to various morphological alternations like significant changes in TSH.	(131) (57)
Mercury	Rats and Mice	Decreased Luteinizing hormone-releasing hormone [LHRH], changes in hypothalamic neuropeptides, decreased Hypothalamic insulin receptor [Insr] mRNA	(132, 133)
Chromium	Rats	Chromium in combination with benzene causes significant alternations in the neuroendocrine and lymphoid systems by disrupting the hypothalamic-pituitary-adrenocortical axis, increased MT-3 mRNA expression in the hypothalamus	(134) (135)

MPs and NPs accumulation have been observed in fish brain tissues, crossing the blood-brain barrier and causing neurotoxic effects (136). MPs also decrease hypothalamic kisspeptin level in zebrafish, responsible for their reproduction (137), and interferes with the HPT axis that distorts their gene expression (138).

Plastic additives like BPA and BPS cause decreased hypothalamic neurons and neuroendocrine disruption (139) while polybisphenols disturb the expression of HPT-axis genes (140). Mice exposed to BPA have shown astrocyte activation and various inflammatory actions in the hypothalamus through activation of the toll-like receptor [TLR4], a receptor that plays a vital role in inflammatory responses in the central nervous system (128). The hypothalamic inflammation induced by BPA impairs the function of proopiomelanocortin [POMC] neurons in the hypothalamic arcuate nucleus [ARC] (128). BPA also alters regulatory and inhibitory responses in the HPG axis and its chronic exposure increases expression levels of GnRH1, Kiss1, and FSH in exposed male and female rodents (141). Similarly, BPA induces increased anteroventral periventricular nucleus [AVPV] Kiss1 neurons in male offspring and enhanced Kiss1 cell number in the rostral periventricular area of the third ventricle of female offspring (126).

Phthalates cause hormonal imbalance by interacting with nuclear receptors, hormonal receptors, signaling pathways, and modulate gene expression linked with reproduction thereby, disrupting the HPG axis that affects fertility (142, 143). Due to HPG axis disruption, phthalates alter the levels of GnRH by interacting with genes of G protein-coupled receptors [GPCRs] on pituitary cells and disrupt FSH and LH ratio by interfering with their receptors on Leydig cells, which consequently disrupt the normal activity of steroidogenic enzymes and steroid hormones (142). DEHP exposure induces early puberty in female rats by inducing upregulation of hypothalamic insulin-like Growth Factor-1 [IGF-1] expression which alters normal hormonal levels of growth hormone [GH] and IGF-1 in the hypothalamus (129). DEHP also adversely affects mRNA and protein expression of KiSS1, GPR54, and GnRH in the hypothalamus thereby interfering with the hypothalamic regulatory mechanism affecting normal gonadal development and hypothalamic hormonal balance in pubertal rats (129).

Similarly, nonylphenol disrupts the negative feedback mechanism of the hypothalamic-pituitary-adrenal [HPA] axis by inhibiting estrogen binding with its receptor (28). TBT disrupts the functional ability of the female HPG axis reducing hormones like hypothalamic GnRH and decreasing the secretion of pituitary LH (125). These abnormalities induced by TBT are associated with dysregulated ovarian steroidogenesis, irregular folliculogenesis, oxidative stress, fibrosis, and abnormal alternations in estrogen and testosterone levels (144). Furthermore, TBT is capable of reducing thyroid follicles, distorting gene expression in the HPT axis and provoking thyroid homeostasis to various morphological alternations like changes in TSH and FT4 levels (57).

Mercury exposure causes adverse alternation in the circulating level of some hormones like FSH, LH, inhibin, and androgen, causing reproductive disruption (121, 145) through its pathogenic changes in the HPA axis and HPG axis (146). Studies found a higher concentration of HPA axis hormones in people exposed to heavy metals (147). Some heavy metal present in electronic waste of electronic devices alters the HPA axis and increases the secretion of corticotropin-releasing hormone [CRH], and adrenocorticotropic hormone [ACTH] (147). Lead (Pb) distorts the mechanism of neurotransmission (148) in the brain and causes variations in the regulation of hypothalamic neurotransmitters that affect the functional ability of gonadotropic hormones by influencing the control of the HPG axis (121, 149). Chromium is a neurotoxicant that increases GR activity and metallothionein isoform 3 [MT3] in the hypothalamus (150). Chromium in combination with benzene causes alternations in the neuroendocrine and lymphoid systems by disrupting the hypothalamic-pituitary-adrenocortical axis (134).

## 6 Effects of MPs, NPs, and their associated chemicals on the pituitary gland

The pituitary gland is a neuroendocrine organ having an essential role in major physiological functions such as growth, sexual development, metabolism, and stress responses (151). Through various regulatory axes, the hypothalamus and pituitary gland regulate neuroendocrine actions including the HPT axis, HPG axis, HPA axis, and HP-somatotrophic axis (126). The hypothalamus-pituitary [HP] axis plays a major integrative role in controlling the mammalian endocrine system. It maintains a balanced homeostatic condition and is responsible for essential hormone secretion that regulates the thyroid, adrenal gland, gonads, somatic growth, and many other functions (126). The hormones secreted by the hypothalamus play a major role in controlling pituitary functions such as metabolism, lactation, growth, and milk secretion (126). Pituitary glands consist of two lobes, the anterior pituitary, and posterior pituitary, linked by the intermediate lobe. The hormones secreted by the anterior pituitary gland into the bloodstream are adrenocorticotrophic hormone, follicle-stimulating hormones, luteinizing hormones, thyroid-stimulating hormone, growth hormone, and prolactin while the posterior pituitary releases oxytocin and antidiuretic hormone (152).

There is a dearth of studies regarding the harmful effects of MPs on the pituitary and we did not find out any research data that have explored the MPs toxicity on the mammalian pituitary gland. However, there is some evidence on the dysregulation of hypothalamic-pituitary axes caused by MPs and their composite EDCs (126, 127) like the HPT axis (138) and HPG axis (127) (**Table 8**).

**Table 8:** The effects of [MPs] additives on the mammalian pituitary gland.

Endocrine disrupter	Species	Harmful effects	References
**Phthalates**	Rats	Altering levels of GnRH, LH, and FSH, increases corticosterone and ACTH levels	(68) (142) (85)
**Bisphenols**	Rats	Effect the pituitary directly by altering its response to TRH released by the hypothalamus	(153)
**PBDEs**	Rats	Significantly alter TH balance at multiple stages of HPT-axis thereby, disrupting normal HPT-axis, and exerting its carcinogenic effects in the pituitary of male rats and the uterus of female rats	(154) (155)
**TBT**	Rats	Decreased secretion of GnRH and LH.	(144)
**HBCD**	Rats	degeneration of the adrenal cortex	(59)
**Organophosphate**	Rats	Decreases fertility by affecting the pituitary gonadotrophins, Cortical hypertrophy of zona fasciculate	(156) (157)
**Mercury**	Human	Inhibits LH and FSH secretion, menstruation disorders, Leydig cells deformation, impaired follicular development	(158) (159)
**Lead [Pb]**	Rats	Suppressed serum FSH	(160)
**Chromium**	Rats	Increased superoxide dismutase activity in the anterior pituitary, oxidative stress in the pituitary gland	(135)
**Cadmium**	Rats	Decreased circulating levels of LH and FSH	(161)

HP axis is vulnerable to a variety of MPs composite EDCs (105) such as BPA (162), PCBs, PBDEs, PBBs, dichlorodiphenyltrichloroethane [DDT] (163), and TBT (164) (**Table 8**). Extensive use of these EDCs in synthetic products, as well as their incorrect disposal, results in a range of environmental contamination, leading to endocrine disruption (126). The consequences of EDCs carried by MPs and NPs (105) on the pituitary gland are the induction of a non-cancerous pituitary tumor known as prolactinoma and stimulation of pituitary hormones like prolactin and TSH (165). The minimal amount of estrogen required to induce tumor is far higher than the normal level so, it is questionable and doubtful whether weak estrogenic disrupters might act as carcinogenic in the pituitary gland (165).

BPA as an essential additive (126) disrupts the regulatory mechanism of the HPT axis through altered TSH levels and affects the pituitary directly by altering its response to Thyrotropin-releasing hormone [TRH] released by the hypothalamus (153).

Mice studies have shown a significant decrease in the expression of pituitary Esr1 with reduced hypothalamic Esr1 expression in rats exposed to 10mg/kg/day diisopentyl phthalate (85). PBDEs as flame retardants alter TH balance at multiple stages of the HPT-axis (154, 155) and exert carcinogenic effects in the thyroid and pituitary of male rats and the uterus of the female rats (154).

Mercury bio-accumulates in the pituitary and thyroid glands and causes endocrine toxicity by altering HP thyroid/gonadal axis (158). Cadmium and arsenic, among the most harmful EDCs, adversely affect the endocrine by altering the secretion of hormones (166).

Mercury inhibits pituitary gland LH and FSH secretion, causing spermatogenesis and sperm count disruption in males and ovarian dysfunction and dysregulated menstruation in females (146). Both Cadmium and arsenic exert xenoestrogenic effects on the interior part of the pituitary gland and reduce LH secretion (167). Arsenic causes neurological abnormalities, and increases mRNA expressions of genes responsible for oxidative responses thus, inducing oxidative stress and apoptosis (168). Similarly, the combined exposure of Pb and cadmium affects the LH and FSH levels in proestrus rats while Pb exposure alone causes a reduction in the fluidity of the pituitary membrane (94).

## 7 Effects of MPs, NPs, and their associated chemicals on the adrenal gland

The adrenal gland is an essential endocrine gland located on the top of the kidneys and composed of the adrenal cortex and adrenal medulla that release hormones like cortisol, aldosterone, epinephrine, and norepinephrine (169). Like the hypothalamus and pituitary, we did not find any research-based analysis about the direct consequences of MPs on the mammalian adrenal gland, except the findings of Stojanović et al. (170) that have identified an increase in the relative weight of rat’s adrenal gland. However, in zebrafish, PS-NPs affect glucose homeostasis by increasing cortisol secretion and such alternation in cortisol levels causes behavioral changes by interfering with brain cells’ electrical activity that alters important molecules like neurotransmitters, enzymes, and receptors (171).

Toxicological findings have recognized the adrenal gland as the most sensitive organ to EDCs because of the critical role of glucocorticoids secreted by the adrenal cortex in maintaining homeostasis (172) (**Table 9**). EDCs may disrupt HPA (186), which induces stress responses causing altered behavioral, neuronal, and immune functions while other abnormalities associated with disrupted HPA axis include anxiety, metabolic disorders, and post-traumatic stress disorder [PTSD] (187).

**Table 9:** The effects of [MPs] additives on the mammalian pituitary gland.

Endocrine disrupter	Species	Harmful effects	References
**BPA**	Rats	Increases the adrenal gland weight in offspring Stimulates a high level of plasma corticosterone by elevating steroidogenic acute regulatory protein (StAR) concentration Exerts adverse consequences on adrenal cell proliferation through ERβ and SHH signaling mechanism, activating cyclin D1 and cyclin D2	(173) (174)
**Phthalates**	Rats	Results in decreased expression of angiotensin II in the adult adrenal gland, reducing aldosterone levels	(172) (175)
**PBDEs**	Rats	4-bromodiphenyl ether (BDE3) increases serum aldosterone and corticosterone levels. It also up-regulates Cyp11b1 expression and causes AMPK signaling disruption by decreasing its phosphorylation	(176)
**TBT**	Mice	Increases intracellular storage and causes the accumulation of lipids and cholesterol in adrenal cells, which results in weakened cholesterol utilization and increased cholesterol levels	(177) (178)
**Organophosphates**	Rats	Isopropylated triphenyl phosphate [IPTPP] causes hypertrophy of the adrenal cortical and increased relative weight of the adrenal glands	(156)
**Phenols**	Rats	Damages the endogenous estrogenic cascade in the adrenal gland, cause changes in the regions of the cortex medulla, Causes cytoplasmic decomposition in cells of the cortex and hemorrhage in the tissue interface	(179)
**DDT**	Rats	Decreased level of catecholamines, norepinephrine, and epinephrine, Impaired aldosterone secretion, reduced the size of zona glomerulosa	(180) (181)
**Mercury**	Human	Alter the metabolism of catecholamines in the medulla of the adrenal gland leading to an elevated level of plasma nor-adrenaline with aging, pathogenesis of hypertension, and metabolic syndromes. lower corticosterone level	(182) (183)
**Chromium**	Rats	Increased adrenal Δ53β-hydroxysteroid dehydrogenase [HSD] activity, adrenal weight, and serum corticosterone level	(184)
**Nickel and cobalt**	Rats	Increased mass of fascicular zone and secretion of glucocorticoids	(185)

BPA as EDC plays an essential role in the development of non-functional adrenal incidentaloma [NFAI] (174) and causes increased adrenal gland weight in offspring of both male and female rats when exposed to food containing BPA of 25mg/kg (173). BPA causes high level of plasma corticosterone by elevating steroidogenic acute regulatory protein [StAR] concentration (174) and altering adrenal cell proliferation through ERβ and Sonic Hedgehog Signaling [SHH], activating cyclin D1 and cyclin D2 (188). BPA also reduces the immunoreactivity of smooth muscle actin [SMA] in smooth muscles of the adrenal capsule and alters the immunoreactivity of adrenal contractile proteins in rats (189). It has been observed that BPA induces a considerable increase in the adrenal index, vascular congestion, cellular destruction, reduced antioxidant enzymes, and decreased expression of vimentin proteins as well as alpha-smooth muscle actin (189). DEHP as an essential plasticizer is associated with decreased expression of angiotensin II in the adult adrenal gland, reducing aldosterone levels (172). Postpartum exposure to 300 mg/kg of DEHP significantly reduced corticosterone levels while 500 mg/kg of DEHP increases corticosterone and ACTH levels and 10 mg/kg of DEHP triggers glucocorticoid receptor [GR] in the HPA axis, resulting in anxiety-like behavior in premature rats (175). PBDEs, such as 4-bromodiphenyl ether [BDE3] increased the level of serum aldosterone and corticosterone (176). BDE3 up-regulates Cyp11b1 expression and causes AMPK signaling disruption by decreasing its phosphorylation in rats exposed to 200mg/kg of BDE3 (176). TBT is an oxidative endocrine disrupter, which increases intracellular storage (177) and causes the accumulation of lipids and cholesterol in adrenal cells, which results in weakened cholesterol utilization and increased cholesterol levels (178). Organophosphates like isopropylated triphenyl phosphate [IPTPP] cause hypertrophy of the adrenal cortical in zona fasciculate and increase the relative weight of the adrenal gland (156).

Nonyl phenols and octylphenol derived from ethoxylates (190) act as an ED, damaging the endogenous estrogenic cascade in the adrenal gland. Nonylphenol causes adrenal disruption by decreasing the noradrenaline cells leading to lethargy and altering stress response in the body (28). While octyl phenol causes changes in the regions of the cortex medulla, cytoplasmic decomposition in cortex cells, and hemorrhage in the tissue interface of pregnant rats (179). DDT, a widespread ED causes cell atrophy and degenerative effects in the adrenal cortex mainly in the zona fasciculate and zona reticularis (191). DDT can bio-accumulate in the thymus, brain, and even in adipose tissue, and induces impairments of both cortex and medulla of the adrenal gland, disrupting hormonal secretion in cortical and chromaffin cells as well as suppressing the thyroxine hydroxylase production in chromaffin cells (191).

The adrenal gland is also vulnerable to heavy metals like mercury, cadmium, cobalt, and copper that affect the zona glomerulosa of the rat adrenal gland (192) and dysregulate HPA axis, altering the hormonal secretion of corticosterone in response to various stressors and interfering with steroid hormones metabolism (183). Among these toxic metals, mercury alters the metabolism of catecholamines in the adrenal medulla, leading to an increased level of plasma nor-adrenaline with aging. Its chronic exposure is associated with the pathogenesis of hypertension and metabolic syndromes (183).

## 8 Conclusion

The abundance and distribution of MPs derived from plastic degradation across the globe are so extensive that we can claim of living in a plastic world. Endocrine toxicity induced by MPs is an emerging issue, despite the subject being rarely documented, there is growing evidence for ingested MPs bioaccumulation in mammalian tissues and organs with deleterious outcomes including endocrine abnormalities, reproductive toxicity, gut microbiota dysbiosis, and defective immunological responses in rodents, rats and mice. Various EDCs or toxic chemicals present in plastic as an additive or adsorbed by MPs enter the body easily, acting as agonists or antagonists for a wide range of hormonal receptors, and induce endocrine toxicity. The identification of adverse consequences of MPs on the mammalian endocrine system is a great challenge due to their rising levels in both terrestrial and aquatic ecosystems. However, there are still no conclusive research reports that have determined the direct consequences of MPs and NPs on the hypothalamus, pituitary, and adrenal gland. So further research studies are essential to be performed to determine the potential hazards of MPs and NPs to regulate laws that reduce exposure to these small plastic particles.

## Author contributions

Conceptualization, SU (1st author) and GN. methodology, SU (1st author) and GN. writing—original draft preparation, SU (1st author), SaU, and GN. writing—review and editing, SU (1st author), SA, XG, SaU, SU (5th author), GN. funding acquisition, XG, KW. All authors contributed to the article and approved the submitted version.
